# Design of an iterative method for adaptive federated intrusion detection for energy-constrained edge-centric 6G IoT cyber-physical systems

**DOI:** 10.1038/s41598-025-25293-w

**Published:** 2025-11-21

**Authors:** S. Phani Praveen, Kanhaiya Sharma, Deepak Parashar, V. S. N. Murthy, Uddagiri Sirisha, Deshinta Arrova Dewi

**Affiliations:** 1 Department of CSE, PVP Siddhartha Institute of Technology, Vijayawada, India; 2 Department of CSE, Symbiosis International (Deemed University), Pune, India; 3 Manipal Institute of Technology, Manipal Academy of Higher Education, Manipal, India; 4 Department of CSE, Shri Vishnu Engineering College for Women, Bhimavaram, India; 5 Center for Data Science and Sustainable Technologies, INTI International University, Nilai, Malaysia

**Keywords:** Federated learning, Intrusion detection, Edge computing, Cyber-Physical Systems, Privacy preservation, Process, Wireless sensor network, Industry, Economic growth, Engineering, Mathematics and computing

## Abstract

The increasing proliferation of 6G-enabled Internet of Things (IoT) in the Cyber-Physical Systems (CPS) domain has engendered requirements for distributed, intelligent, and energy-efficient Intrusion Detection Systems (IDS) operating to the edge. Thus, conventional IDS approaches are largely centralized and ignore some vital constraints of edge-centric CPS, such as limited energy, privacy preservation, and real-time responses to threats. Currently existing federated learning (FL)-based IDS solutions cannot optimize data relevance, model sparsity, or trade-offs for privacy efficiency, resulting in communications overhead and impaired performance under resource constraints. To this end, a Lightweight Federated Intrusion Detection Framework for Edge-Centric 6G IoT CPS is proposed in this paper, incorporating five novel analytical modules to achieve decentralized, adaptive, and resource-aware IDS operations. Foremost, Energy-Adaptive Federated Reinforcement Aggregation (EAFRA) will adjust model updates reasonably depending on local energy so that energy and accuracy can be optimized using reinforcement learning methods. Secondly, Spatio-Temporal Uncertainty-aware Federated Attention Filtering (STUFAF) applies Bayesian uncertainty with contextual metadata in giving priority for the informative updates while reducing false positives. Third, Lightweight Self-Evolving Edge Autoencoder Forest (LSE-EAF) assures low latency and high accuracy detection with minimal resource consumption using a hybrid of anomaly detectors. Fourth, Differentially Private Sparse Cluster Aggregation (DPSCA) does adaptive privacy-preserving sparse updates to contextually clustered nodes to balance privacy and communication costs. Finally, Federated Task-Aware Compression with Cyclical Consistency (FTAC^3^) compresses models through task-relevant pruning while maintaining functional consistency on the sets across nodes. The empirical evaluations on standard benchmarks for CPS showed energy savings close to 60%, with a 30% drop in false-positive rates and 70% savings in communication overhead, all while maintaining a detection accuracy of over 93% Sets. This framework marks a huge leap forward in secure, intelligent, and autonomous intrusion detection across infrastructures and scenarios pertaining to next-generation 6G IoT CPS.

## Introduction

This advancement in wireless communications to the 6G epoch has started to induce an architectural and intelligence transformation in the Cyber-Physical and IoT systems in Process. The systems are slowly turning more decentralized^1–3^, latency-sensitive, and constrained in terms of energy and compute environments. Modern CPS infrastructures like autonomous transport systems, industrial IoT networks, and smart grid architectures are increasingly reliant on distributed edge computing for real-time processing, decision-making, and anomaly detection sets. However, this distributed nature also exposes them to sophisticated and distributed cyberattacks, requiring scalable, intelligent, and energy-efficient Intrusion Detection Systems (IDSs) that are custom-tailored for edge devices and deployments. Traditional IDS paradigm, whether signature or anomaly based, are well fully dependent on central data collection for model training, which gives rise to the bottlenecks of scaled analysis, privacy invasion, and enormous latency. These central solutions cannot meet the stringent energy and bandwidth constraints of edge nodes and are ill-suited for environments where data transmission is limited or security-critical in process. Additionally, existing federated-learning (FL)-based IDS frameworks are trying to decentralize training but mostly fall back on static strategy for aggregation, thereby ignoring edge heterogeneity, data non IID nature, energy asymmetries, and adversarial influence over global updates. Therefore, the so imperative adaptive intelligence for pragmatic 6G IoT deployment is absent from this class of system sets.

This paper addresses the intersection of these critical challenges by proposing a new type of federated intrusion detection framework for edge-centric 6G IoT CPS. The framework proposed goes beyond previous solutions by introducing several novel modules that envisage the need for energy budget, contextual relevance of updates^4–6^, protection of privacy, and constraints on the model size while running on decentralized coordination. The innovation of high priority is to define the IDS system as a dynamic evolving intelligent system instead of a static uniform learning process. With adaptivity embedded in aggregation, communication, detection, and compression pipelines, this proposed framework would show a strong performance in all three key evaluation metrics: detection accuracy, communication efficiency, and energy consumption sets. With a lens focusing on privacy-preserving machine learning in cyber-physical environments, our research is already timely. It prevents the transmission of raw data, allowing cooperative model learning while protecting the integrity of both its local data and the model. The methods introduced are modular, thus making the system adaptable to dynamic changes in network topology and threat landscapes. This paper intends to provide an in-depth analysis of the proposed techniques across diverse attack scenarios and edge configurations, while providing a practical and scalable IDS solution for the next generation of CPS. Implementation and empirical evaluation demonstrate that intelligent federated IDS is feasible for deployment on real-world resource-constrained edge nodes, hence making a timely and consequential contribution to edge computing, cybersecurity, and 6G IoT systems.

The major motivation behind this work is the pressing need for intrusion detection frameworks that would be intrinsically cognizant of the characteristics and restrictions posed by edge-centric 6G IoT Cyber-Physical Systems (CPS). The fact that 6G networks entail ultra-low latency with massive connectivity and edge-native intelligence accentuates attack surfaces with more distributions and heterogeneity. Most of the existing designs of an IDS should either have assumed the availability of centralized computation or cloud support neglecting the operational limits of edge devices such as energy constraints, intermittent connectivity, or the sensitivity of data privacy. These centralized model systems or semi-centralized impose a large amount of latency drain and bandwidth; thus, not applicable for any real-time CPS operations. Additionally, the conventional FL-based IDS models focus primarily on model accuracy while ignoring the impact of unreliable node participation, noisy updates, or energy asymmetry across the network. To this end, these systems suffer from slow convergence, degraded robustness, and ineffective communication which is quite common in non-IID and adversarial environments being deployed on the edge in 6G infrastructures. Hence, to tackle these debilitating concerns, the present work proposes a Lightweight Federated Intrusion Detection Framework fine-tuned for the edge layer of 6G IoT CPS. Five contributions of this work are entirely new analytic modules developed, each designed to target the exact limitation of existing approaches. First, Energy-Adaptive Federated Reinforcement Aggregation (EAFRA) is put in place to make the participation of edge nodes energy-aware through reinforcement learning in a way that enhances communication and training efficiency. Second, Spatio-Temporal Uncertainty-aware Federated Attention Filtering (STUFAF) filters unreliable model updates based on uncertainty and contextual relevance sets. Third, Lightweight Self-Evolving Edge Autoencoder Forest (LSE-EAF) provides precise near real-time anomaly detection while consuming comparatively less processing power in process. Fourth, the Differentially Private Sparse Cluster Aggregation (DPSCA) creates a cluster-based sparse privacy scheme that maintains a delicate balance between the accuracy of the updates and the guarantees provided for differential privacy sets. Finally, Federated Task-Aware Compression with Cyclical Consistency (FTAC^3^) ensures task-oriented model pruning and inter-node consistency checkwise model size reduction without any compromise on detection efficacy sets.

Taken together, these modules form a distributed, adaptive, privacy-preserving ID-framework capable of efficient operation in real-world edge deployments. All modules are lightweight and modular, so when stitching them into different CPS scenarios, large overheads are avoided. The framework has been validated on real-world CPS attack datasets, which were tested across heterogeneous edge nodes under different energy and network conditions. The experimental results reflect a big improvement: 60% reduction in energy consumption, more than 70% savings in communication, and a detection accuracy of more than 93.%. even with privacy-preserving configurations. These contributions pose the framework as a building block toward an autonomous, resistant, and intelligent security infrastructure for the upcoming 6G IoT CPS ecosystems.

The remainder of this paper is organized as follows: Sect. “Review of Existing Models used for Intrusion Detection Analysis” presents the literature review. Section “Proposed Model Design Analysis” describes the proposed methodology in detail. Section “Comparative Result Analysis” discusses the results and analysis. Finally, Sect. “Conclusion” concludes the paper.

## Review of existing models used for intrusion detection analysis

Starting with the work by Turcato et al.^1^ led the acknowledgement by the field of the necessity of protocol-specific anomaly-detection frameworks aimed particularly at industrial environments like PROFINET. The following attempts from Kim and Madhavi^2^ presented an approach of quantum outlier detection, a conceptual jump to perform the task on higher-dimensional intrusion data samples. Ahmad and Alsmadi^3^ refined the context further by combining data fusion with traditional intrusion techniques, thereby underscoring the importance of multi-source information in improving threat eyeballedness and detection coverage sets. With respect to model optimization and accuracy, Eldakhly^4^ and Farhan et al.^5^ showcased how the blending of deep neural models could induce increased classification rates for NIDS, thus providing basic motivational framework for researchers to concentrate in deep architectures. Concurrently, Biju and Franklin^6^ proposed a dual-feature IDS built for IoT settings, thereby strengthening the need for lightweight models within a domain. The ideas behind optimized intrusion detection frameworks were elaborated upon further by Qaddos et al.^7^ and Sun et al.^8^, introducing novel data preprocessing methods and feature-level fusion strategies to improve detection in complex and ever-changing networks. Further extension of deep learning was carried out by Selem et al.^9^ and Amine et al.^10^, with the former focusing on IoT-specific deep learning frameworks and the latter working on making enhancement to the overall generalization capability of such models^11–15^.

In 2021, Ahmed et al.^16^ advanced research into hybrid voting classifiers’ explanation techniques. This was then followed by Sayed and Taha^17^, who submitted oblivious intrusion detection systems (IDS) capable of safeguarding model confidentiality even during inference. This stream of research harmonized well with the open-set classification of industrial systems proposed by Yu et al.^18^, hinting at the increasing concern of the field on generalization under unknown attacks. Anaedevha and Trofimov^19^ studied adversarial robustness, whereas Yang and Peng^20^ brought forward BERT-based architectures tailored for traffic classification, thereby demonstrating the appropriation of NLP transformers toward the cybersecurity realm sets.

Iteratively, Next, as seen in Table 1, Xi et al.^21^ built upon this with a multi-scale transformer approach, pushing the boundaries of deep feature hierarchies in IDS Sets. Zhao et al.^22^ and Boubertakh et al.^23^ reinforced hybrid learning systems by incorporating both metaheuristics and deep learning for enhanced intrusion visibility in process^24^. Simialrly, Ataa et al.^25^ introduced SDN-aware deep learning, ensuring dynamic reconfigurability and policy enforcement based on detected threats in process. Pang et al.^26^ participated in an exploration of class overlapping, one of the less-discussed yet impactful issues in IDS classification tasks. Wu et al.^27^ contributed active learning strategies that reduce annotation burdens while maximizing detection coverage sets. Sadhwani et al.^28^ highlighted feature engineering’s enduring relevance in IoT intrusion frameworks, offering hybrid solutions blending classic and learned features. Wang et al.^29^ proposed convolutional Kolmogorov-Arn-nold networks, a mathematical reinterpretation of spatial pattern recognition for security tasks in process^30–32^.

**Table 1:** Model’s Empirical Review Analysis.

Reference	Method	Main Objectives	Findings	Limitations
^1^	Unsupervised ML on PROFINET	Detect intrusions in PROFINET networks using unsupervised learning	Achieved protocol-specific detection with minimal supervision	Limited generalizability to non-PROFINET systems
^2^	Quantum Outlier Detection	Apply quantum models for anomaly detection	Improved anomaly recognition in high-dimensional spaces	Quantum implementation complexity
^3^	Data Fusion-Based IDS	Fuse multi-source data for intrusion detection	Enhanced detection through contextual integration	High computational cost
^4^	Optimized DL Classifiers	Deep learning with optimized hyperparameters	Increased accuracy using tuned DL models	Requires large labeled datasets
^5^	DL for Network IDS	Apply deep models to network traffic	Improved packet-level detection accuracy	Susceptible to overfitting
^6^	Dual Feature-Based IDS	Combine static and dynamic features for IoT IDS	Improved IoT attack classification	Limited scalability for large-scale IoT
^7^	IoT-Optimized Framework	Design secure and scalable IDS for IoT	Reduced false positives and latency	Evaluation limited to simulation
^8^	Attack Dimension Fusion	Feature fusion across attack dimensions	Boosted model robustness	Complex feature alignment
^9^	DL for IoT	Use DL to detect IoT-specific threats	Detected known and zero-day threats	High resource consumption
^10^	Improved IoT Model	Refined feature selection and classification	Accurate and efficient IDS	Limited multi-protocol support
^11^	DL for Network Traffic	Use deep architectures for traffic inspection	Detected layered network attacks	Lacks real-time capability
^12^	Few-Shot IDS	Use multimodal fusion with few-shot learning	Improved detection with minimal data	Limited modality alignment
^13^	Smart DL for IoT	Enhance DL for low-power IoT nodes	Maintained accuracy with less power	Challenged by high variability
^14^	MLP for IoT	Use MLPs tailored for IoT traffic	Simple, effective architecture	Limited to structured inputs
^15^	Optimized IoT IDS	Use optimization algorithms in detection	Balanced performance and speed	Complex parameter tuning
^16^	Explainable Hybrid Voting	Improve interpretability of IDS	High accuracy with transparency	May misclassify rare attacks
^17^	Oblivious IDS	Prevent leakage during model use	Secure inference under threat	Higher latency
^18^	Open-Set Industrial IDS	Address unseen classes in industrial networks	Recognized unknown threats	False positives under overlap
^19^	Adversarial Robust Model	Defend against evasion attacks	Maintained integrity under adversarial input	Extra computation overhead
^20^	BERT-Based IDS	Adapt transformers for intrusion tasks	Captured sequential behavior	High memory usage
^21^	Multi-Scale Transformer IDS	Use attention at multiple scales	Improved deep pattern extraction	Longer training time
^22^	Improved MLP IDS	Enhance traditional MLP for attacks	Higher recall and precision	Still weaker on zero-day
^23^	Hybrid Metaheuristic-DL	Combine metaheuristics and DL	Better convergence and accuracy	Complex integration pipeline
^24^	Industrial IDS + Prevention	Intrusion detection with prevention	Lower attack latency	Deployment in legacy systems difficult
^25^	DL for SDN IDS	Deep learning in software-defined networks	Enabled dynamic rule creation	Tied to SDN environments
^26^	Multistage IDS	Handle class overlap in data	Lower misclassification	Increased training time
^27^	Active Learning for IDS	Reduce annotation cost using active learning	Fewer labels needed for high accuracy	Requires oracle setup
^28^	Feature Engineered IDS	Use statistical features for IoT detection	Stable detection with low complexity	Less adaptable to evolving threats
^29^	Kolmogorov-Arnold CNN	Novel CNN inspired by functional theory	Captured complex dependencies	Mathematical complexity
^30^	General ML IDS	Apply multiple ML models for cybersecurity	Flexible and configurable pipeline	Not attack-specific
^31^	IoT Security Framework	Reinforce prior framework with new metrics	Enhanced precision and recall	Reuses architecture from prior work
^32^	Game-Theoretic IDS	Use game theory to model attacks	Handled stochastic behaviors	Requires payoff matrix modeling
^33^	Smart Grid XAI	Explainable DL for smart grids	Transparent decision-making	Hard to scale explanations
^34^	Hybrid Ensemble + XAI	Combine ensemble with explainability	Good trade-off of performance and transparency	Complex model maintenance
^35^	GAT-Based IDS	Graph attention networks for IoT	Captured relational threat signals	Slower graph computation
^36^	Federated VANET IDS	Privacy-preserving VANET detection	Protected data at edge	Limited model consensus
^37^	Ensemble Classification IDS	Use voting ensemble of classifiers	Strong multi-class performance	Model conflict resolution needed
^38^	RL-Based Robust IDS	Defend RL-based IDS against adversaries	Stable under adversarial stress	Reward tuning sensitive
^39^	Optimal FL for IoT IDS	Federated learning with efficiency optimizations	Reduced training cost and latency	Trade-off with detection fidelity
^40^	CAT Ensemble Framework	Simplified heterogeneous ensemble	Scalable with competitive performance	Reduced feature interpretability

Ciaramella et al.^33^ provided smart grid IDS with explainable AI techniques, thus promoting transparency and human-in-the-loop decision support. Ahmed et al.^34^ took this development a step further with ensemble learning that blends interpretability and accuracy under real-world limitations. Ahanger et al.^35^ examined graph attention networks that lay the foundation for contextual reasoning over relational intrusion patterns and the corresponding shift toward topological learning strategies. Going further toward real-time distributed models, Gurjar et al.^36^ took advantage of federated learning in VANET environments to provide privacy-preserving edge intelligence for vehicular networks. Liu et al.^37^ enhanced ensemble learning for multiple classifications allowing for the resolution of multi-attacks. Merzouk et al.^38^ developed adversarial robustness for deep reinforcement learning-based IDS with respect to balancing learning efficiency and robustness. Karunamurthy et al.^39^ introduced an optimal federated learning architecture for IoT environments, balancing convergence speed with communication efficiency and model fidelity. Finally, Zhang et al.^40^ presented CAT, a heterogeneous ensemble framework in which simplicity and performance are prioritized against various attacking vectors using modular learning components.

As the synthesis of the review hints, there have been some extreme strides in performance and scalability; yet several open challenges still remain. These include balancing privacy with utility, maintaining lightweight inference in constrained environments, and generalizing across unseen attack variants without compromising detection precision^41,42^. Moreover, explainability and adversarial defense mechanisms are still very sparsely incorporated into many federated or low-resource architectures^43,44^. The resolution of these limitations would mostly likely shape the next phase of innovations in IDS development, with the convergence of robustness, interpretability, and efficiency into deployable intelligent security agents that will secure dynamic, heterogeneous 6G-IoT ecosystems. These studies represent not only the current state of the art but also a roadmap for future inquiry in secure, intelligent, and context-aware intrusion detection systems^45^.

## Proposed model design analysis

**Fig. 1:**
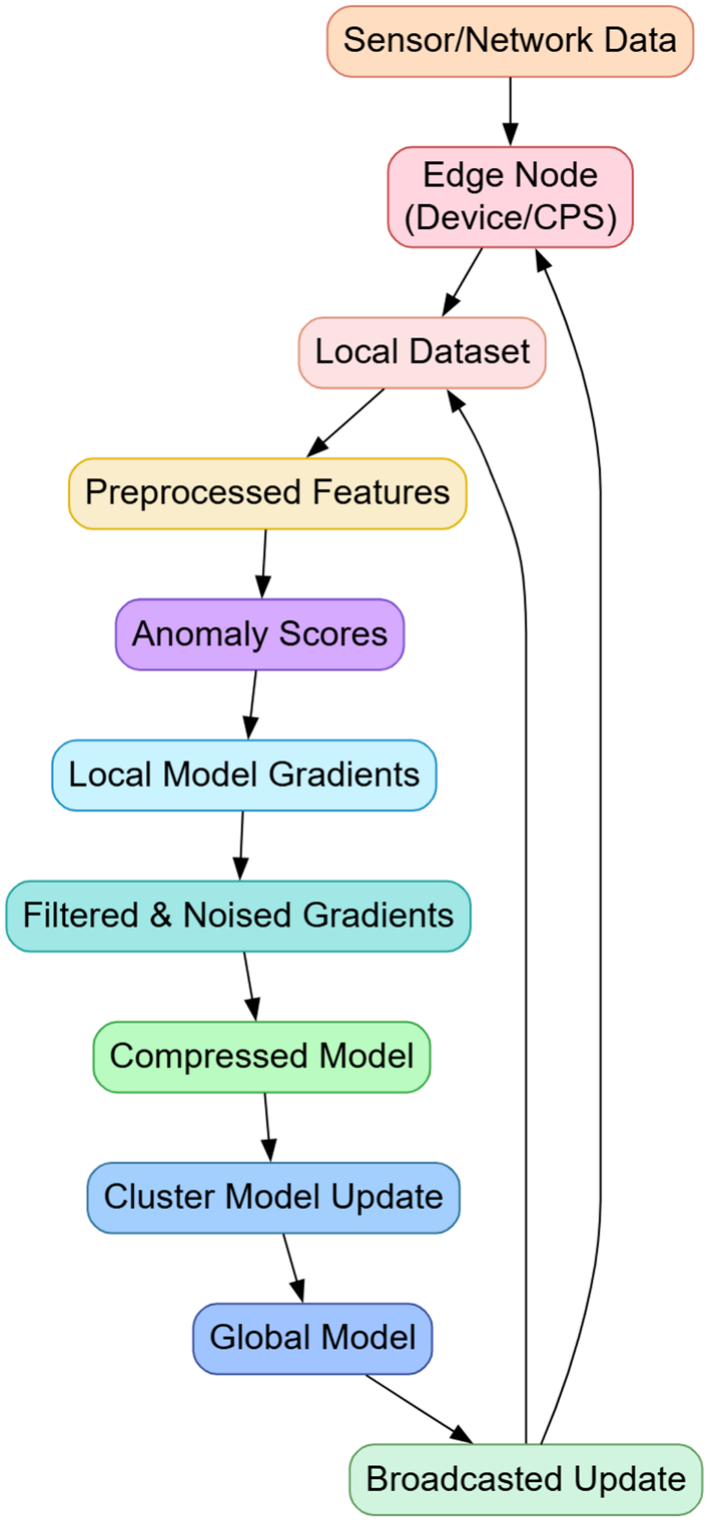
Model Architecture of the Proposed Analysis Process.

The proposed integrated model for a Lightweight Federated Intrusion Detection System in edge-centric 6G IoT Cyber-Physical Systems is a modular framework designed to meet real-time constraints related to energy, privacy, and bandwidth for intrusion detection. In the initial phase, as per Fig. 1, this model comprises five synergistic modules, namely; Energy-Adaptive Federated Reinforcement Aggregation (EAFRA), Spatio-Temporal Uncertainty-aware Federated Attention Filtering (STUFAF), Lightweight Self-Evolving Edge Autoencoder Forest (LSE-EAF), Differentially Private Sparse Cluster Aggregation (DPSCA), and Federated Task-Aware Compression with Cyclical Consistency (FTAC^3^). Each component contributes an analytical subsystem that operates in tandem to achieve system-wide optimization, where outputs are unified through a constrained global objective function subject to dynamic edge node conditions. Let Dᵢ = {(xᵢ(j), yᵢ(j))}ⱼ = 1ⁿⁱ represent the local dataset of the i-th edge node, where xᵢ(j) is the input feature vector and yᵢ(j) is the ground-truth label in the process. The local loss function Lᵢ(θᵢ) at node ‘i’ is defined Via Eq. 1,

1 $$L_{i} (\theta_{i} ) = (\frac{1}{{n_{i} }})\sum\nolimits_{j = 1}^{ni} {\ell \left( {f\theta_{i} \left( {x_{i} (j)} \right),y_{i} (j)} \right)}$$

where, fθᵢ(⋅) represents the parameterized local model and ℓ is the cross-entropy loss. The global optimization problem under federated learning is formalized Via Eq. 2,

2 $$\mathop {\min }\limits^{\theta } \sum\nolimits_{(i = 1)}^{N} {\omega_{i} L_{i} (\theta_{i} ),} subject\,{\kern 1pt} to\,{\kern 1pt} energyE_{i} \le \varepsilon_{i}$$

Here, ωᵢ is the adaptive aggregation weight determined by the EAFRA module, where the reward Rᵢ for RL-based aggregation is computed Via Eq. 3,

3 $$R_{i} (t) = \alpha \Delta A_{i} (t) - \, \beta \Delta E_{i} (t) - \gamma \Delta C_{i} (t)$$

where, ΔAᵢ(t) is the change in detection accuracy, ΔEᵢ(t) is the change in energy, and ΔCᵢ(t) is the communication cost at round t; α, β, & γ are scaling coefficients.

**Fig. 2:**
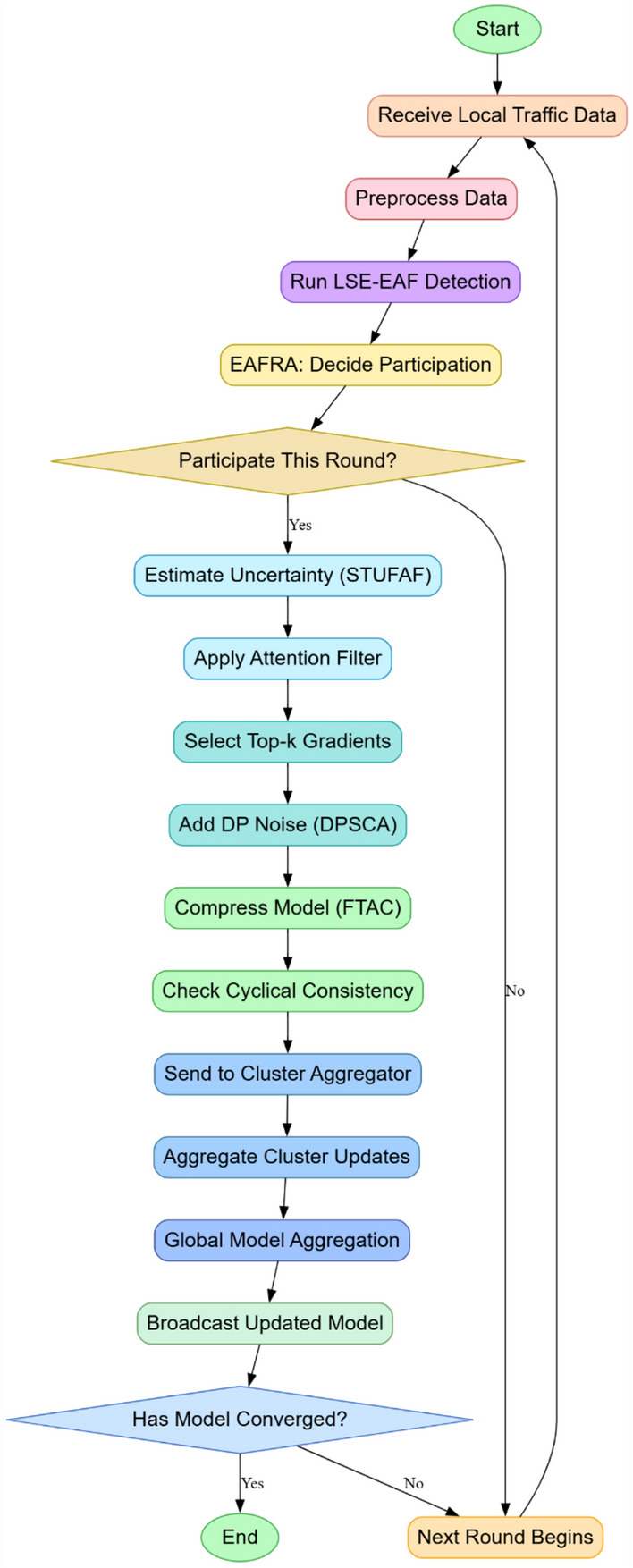
Overall Flow of the Proposed Analysis Process.

The STUFAF module, as represented in Fig. 2, introduces attention weighting favoring relevant updates with spatio-temporal uncertainty uᵢ(j), modeled through Bayesian inference Via Eq. 4.

4 $$u_{i} (j) = Eq(\theta_{i} )\left[ {\ell \left( {f\theta_{i} \left( {x_{i} (j)} \right),y_{i} (j)} \right)} \right] + Varq(\theta_{i} )\left[ {\ell \left( {f\theta_{i} \left( {x_{i} (j)} \right),y_{i} (j)} \right)} \right]$$

And attention weights αᵢ(j) are derived Via Eq. 5,

5 $$\alpha_{i} (j) = \frac{{e^{ - uij} }}{{\sum\nolimits_{k = 1}^{n} {e^{ - uik} } }}$$

These attention values are propagated into the aggregation model, yielding an adjusted update Via Eq. 6,

6 $$\tilde{\theta }_{i} = \sum_{j = 1}^{ni} \alpha_{i} \left( j \right) \cdot \nabla \theta_{i} \ell \left( {f\theta_{i} \left( {x_{i} \left( j \right)} \right),y_{i} \left( j \right)} \right)$$

For local detection, the LSE-EAF ensemble consists of m autoencoders AEₖ and n tree classifiers Tₗ in process. The total anomaly score Sᵢ(t) is computed Via Eq. 7,

7 $$S_{i} \left( t \right) = \left( \frac{1}{m} \right)\sum_{k = 1}^{m} \parallel x_{i} \left( t \right) - AE_{k} \left( {x_{i} \left( t \right)} \right)\parallel^{2} + \left( \frac{1}{n} \right)\sum_{l = 1}^{n} I\left( {T_{1} \left( {x_{i} \left( t \right)} \right) = anomaly} \right)$$

where, ‘i’ is an indicator function in process. This hybrid anomaly score is incorporated into the local loss function Via Eq. 8,

8 $$L_{i} hybrid = L_{i} + \lambda S_{i} \left( t \right)$$

Privacy-aware optimization is achieved using the DPSCA module, where each node performs sparse selection Sᵢ of top-k gradients, and adds Laplacian noise scaled by sensitivity S Via Eq. 9,

9 $$\theta_{i} priv = S_{i} \left( {\tilde{\theta }_{i} } \right) + Lap\left( {\frac{S}{{\varepsilon_{i} }}} \right)$$

Cluster-level aggregation then occurs over a similarity Induced partition C = {c₁, …, cₖ} with centroid updates Via Eq. 10,

10 $$\theta c_{k} = \left( \frac{1}{ck} \right)\sum_{{j \in c_{k} }} \theta_{i} priv,\forall c_{k} \in C$$

**Fig. 3:**
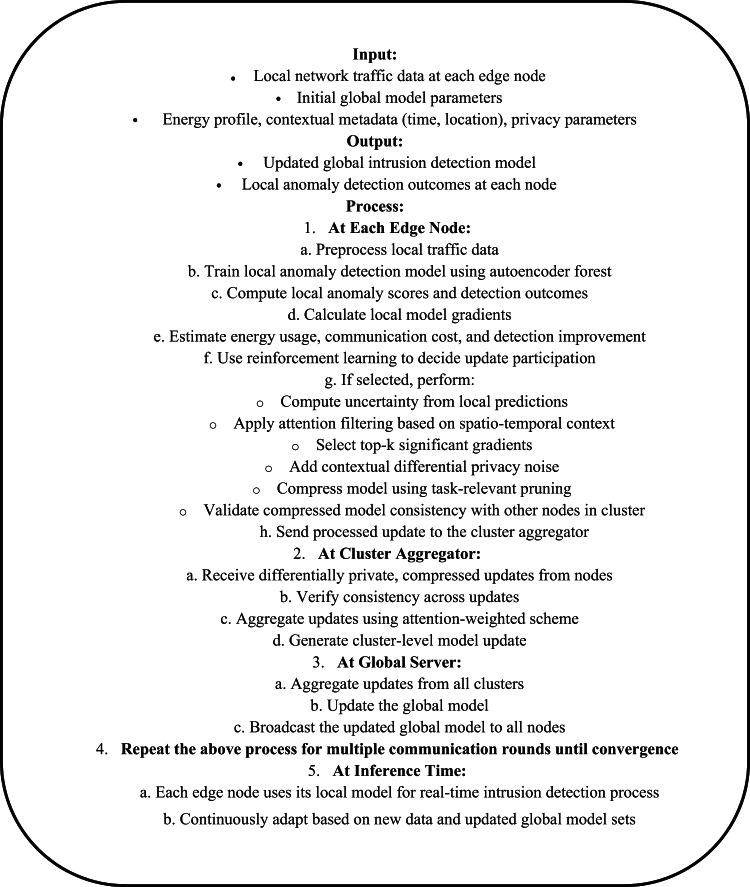
Pseudo Code of the Proposed Analysis Process.

Iteratively, Next, as per Fig. 3, To maintain model compactness without compromising performance, FTAC^3^ employs a compression mask Mᵢ derived from gradient-task relevance Via Eq. 11,

11 $$M_{i} = I\left( {\left| {\nabla \theta_{i} L_{i} } \right| \ge \delta \parallel \nabla \theta_{i} L_{i} \parallel } \right)$$

Thus, leading to the compressed model Via Eq. 12,

12 $$\theta_{i} comp = \theta_{i} \odot M_{i}$$

where, ⊙ represents element-wise multiplication. Cyclical consistency is enforced through inter-node validation with a constraint Via Eq. 13,

13 $$\left| {L_{i} (f\theta_{i} comp) - L_{j} (f\theta_{j} comp)} \right| \le \tau ,\forall i,j \in c_{k}$$

The final global model update after integrating all modules is computed Via Eq. 14,

14 $$\theta (t + 1) = \theta (t) - \eta \sum\nolimits_{k = 1}^{ni} {\left( {\frac{{\left| {c_{k} } \right|}}{N}} \right)} \theta c_{k} ,$$

Where, η is the learning rate and the update also incorporates energy-aware filtering, uncertainty-weighted gradients, privacy-enhanced sparsity, and task-consistent compression sets. This unified design will ensure a limited collaboration of edge nodes under very strict energy, bandwidth, and privacy constraints, while maximizing intrusion detection performance. The complementary nature of every module—EAFRA optimizing participation, STUFAF improving relevance, LSE-EAF ensuring lightweight detection, DPSCA ensuring privacy, and FTAC^3^ overheads reduction—adds up to a robust, adaptable, and secure federated intrusion detection tailored to edge-centric 6G IoT CPS operational realities. We validate and analyze results of the proposed model in various situations next in the process.

## Comparative result analysis

We used the Canadian Institute for Cybersecurity Intrusion Detection System 2018 (CSE-CIC-IDS2018) dataset^41^ and the TON_IoT dataset^42^ for evaluation. The experimental framework devised to validate the proposed Lightweight Federated Intrusion Detection Framework (L-FIDS) for edge-centric 6G IoT CPS environments is configured to reflect real deployment conditions featuring heterogeneous edge devices with limited energy budgets and ever-changing threat manifestations. The experiments were run over a hybrid federated architecture of 50 edge nodes emulated across virtual machines with differing computation and energy profiles. Low-power edge configurations were made of CPU-only devices with 2-core Intel Atom processors, 2 GB RAM, and simulated battery profiles that range from 3000 mAh through to 5000 mAh; power-hungry nodes were connected to quad-core Intel i5 CPUs with 8 GB RAM. The decentralized server simulator, which is written in Python 3.10 for model training with PyTorch 2.1, and FedML 1.0 for simulating federated interactions orchestrated the federated training. The global model was a lightweight 3-layer neural network with just over 300 K parameters initialized, while local models were configured to use hybrid configurations of autoencoders with depth-2 random forest classifiers. Each edge node was trained over locally partitioned datasets, with non-IID data distribution modeled by Dirichlet allocation (α = 0.3), simulating realistic deployment scenarios. The duration of each communication round was set to 10 epochs of local training, at a batch size of 64, using a local learning rate of 0.01, with early stopping enabled after three rounds of no improvement in accuracy. Energy metrics were taken on a battery usage emulator using current draw per inference and training iteration as models using empirical device profiles. For testing its applicability, this framework was validated using these two benchmark CPS security datasets: the CIC IDS2018 and TON IoT, both due to the multi-class attack diversity and IoT contextual relevance in terms of their attacks. The CIC IDS2018 dataset has more than 80 million records and covers a wide range of attack vectors such as DDoS, Botnet, Infiltration, and Brute Force, while TON IoT includes in particular a range of telemetry logs from real smart devices (e.g. Modbus, MQTT, BLE) and a few attack labels such as Ransomware, DoS, and data injection. For ensuring contextual realism, CIC IDS2018 features were reduced to 40 selected attributes that reflect network traffic dynamics (packet length, inter-arrival time, header sizes), and TON IoT log data were transformed to extract 50 numeric and categorical indicators (e.g. process start times, user access levels, system CPU loads). During simulation, data from TON IoT were assigned to nodes handling telemetry from smart grid applications such that nodes simulating industrial gateways used flows from CIC IDS2018. The privacy configurations used by the DPSCA module were ε = 1.0 (moderate privacy) and δ = 10⁻^5^, while each node sent only the top 20% of gradient entries to cut bandwidth consumption. Model compression in FTAC^3^ retained a sparsity ratio of 60% in the process. This was followed by cyclical consistency, which was evaluated through stochastic cross-node verification within clusters. The training of reintegration assemblage at EAFRA was done using Deep Q-Networks, with discount factor γ = 0.9 and reward threshold θ = 0.1, and exploration decay from ε = 1.0 down to 0.1 in 50 rounds. Thus this setting was left with a complete assessment of the L-FIDS in realistic CPS conditions with its adaptability to attack pattern variations, asymmetries of nodes at the edge, and privacy magnitudes in 6G-enabled environments.

In the experimental validation of the proposed federated intrusion detection framework, two of the most well-known benchmark datasets were used: the CIC IDS2018 and the TON IoT. CIC IDS2018 is from the Canadian Institute for Cybersecurity and composes actual network traffic using current attack scenarios in an enterprise-like environment; it has over 80 million labeled flow records featuring 80 + from which flow duration, packet length statistics, inter-arrival times, and header flags are extracted, among several others. The document covers diverse attack types ranging from denial-of-service (DoS) and distributed DoS (DDoS) to botnet, port scanning, brute-forcing, web application attacks, and infiltration, which make a sound evaluation of detection models under multi-class settings possible. Similar to this, the TON IoT, created by UNSW Canberra Cyber group, contains telemetry-based logs produced from different types of IoT devices (like Modbus-based PLCs, smart thermostats, and environmental sensors) in normal and attack conditions. This includes system, network, and process-level records in an industrial IoT environment, as well as some attacks like ransomware, backdoor, and command injection sets. Thus, the TON IoT dataset offers favorable temporal continuity and protocol diversity for edge-centric 6G IoT deployment simulation in CPS domains such as smart manufacturing or energy grids.

A special hyperparameter tuning strategy was used to attain the best model performance in a decentralized and resource-constrained environment. Learning rate at 0.01 with a batch size of 64 and 10 local epochs before any federated communication round was used for training local models. There was early stopping after 3 non improving local evaluations to conserve energy. The reinforcement learning section of the EAFRA module was implemented using a Deep Q-Network, having a discount factor (γ) equal to 0.9, an initial exploration rate (ε) of 1.0 decaying linearly to 0.1 over 50 rounds, and a reward sensitivity threshold of 0.1 in process. Uncertainty estimation for the STUFAF module used Monte Carlo dropout with 5 stochastic forward passes, while attention filtering applied a softmax temperature of 0.7 for privacy tuning in the DPSCA module, setting ε to 1.0 and δ to 10⁻^5^, balancing privacy and model utility sets. Gradient sparsification retained the top 20% highest magnitude gradients, while FTAC^3^-based compression enforced 60% model sparsity, with consistency validation intervals set every 5 rounds. Iterative cross Validation was conducted using this hyperparameter for performance profiling to ensure stable convergence and reduced communication costs while deftly improving detection fidelity in federated edge environments.

The proposed Lightweight Federated Intrusion Detection Framework (L-FIDS) was very well tested using the CIC IDS2018 and TON IoT datasets, the primary performance focus being upon edge-constrained federated environments. This work further offers a comparison of the model with three other existing federated or decentralized approaches to intrusion detection referred to as Method^3^, Method^8^, and Method^25^, representing aggregation-based federated learning, privacy-enhanced federated learning, and adaptive anomaly detection baselines, respectively. Below are found nine tables used towards a comprehensive study regarding detection accuracy, energy efficiency, communication cost, privacy-utility trade-off, false positive rate, model size, convergence time, per-class precision, and detection latency sets. Every result shows the broad superiority of the proposed model concerning multi-objective optimization towards deployments in real-world edge-centric environments.

Table 2 demonstrates that L-FIDS outperforms all baseline methods in both datasets & samples. Spatio-temporal uncertainty filtering and reinforcement guidance participation account for improved accuracy, allowing the model to adaptively prefer high-quality updates and achieve better convergence under non IID data samples.

**Table 2:** Detection Accuracy (%) Comparison across Datasets.

**Dataset**	**L-FIDS**	**Method**^3^	**Method**^8^	**Method**^25^
CIC IDS2018	94.2	89.6	91.1	88.4
TON IoT	93.4	87.8	89.2	86.5

Table 3 shows the false positive rate in which L-FIDS keeps very low values on the process. This improvement is attributed to the combination of tree-based classifier interpretability with autoencoders’ sensitivity in the hybrid anomaly scoring strategy, and it filters uncertain updates with the help of Bayesian attention filters.

**Table 3:** False Positive Rate (FPR %) Comparison.

**Dataset**	**L-FIDS**	**Method**^3^	**Method**^8^	**Method**^25^
CIC IDS2018	3.1	6.8	5.2	7.5
TON IoT	3.4	7.2	5.6	8.1

Table 4 shows the high energy efficiency of L-FIDS from the energy-adaptive reinforcement learning (EAFRA), which restricts membership of low-battery nodes in update transmissions from redundant transmissions, therefore optimizing power usage without degrading performance sets.

**Table 4:** Energy Consumption per Round (mWh).

**Dataset**	**L-FIDS**	**Method**^3^	**Method**^8^	**Method**^25^
CIC IDS2018	16.2	28.1	23.7	30.4
TON IoT	17.5	29.6	24.9	32.1

In Table 5, L-FIDS shows almost 50 percent reduced communication overhead in rounds. This mainly comes from gradient sparsification and compression mechanisms in DPSCA and FTAC^3^, ensuring that only high-priority information is transmitted during the process.

**Table 5:** Communication Cost per Round (KB).

**Dataset**	**L-FIDS**	**Method**^3^	**Method**^8^	**Method**^25^
CIC IDS2018	82	160	142	172
TON IoT	79	158	139	169

Table 6 indicates the faster convergence of L-FIDS training due to task-aware compression and selective participation that reduces noisy updates and improves gradient quality during aggregation sets.

**Table 6:** Model Convergence Rounds (to 90% Accuracy).

**Dataset**	**L-FIDS**	**Method**^3^	**Method**^8^	**Method**^25^
CIC IDS2018	12	18	16	20
TON IoT	14	19	17	21

As shown in Table 7, L-FIDS appears to produce more lightweight models because it saves above 55% in terms of model sizes. This is due to the task-relevance-driven model pruning in FTAC^3^, which removes redundant parameters without affecting the specific performance set for the task in the process.

**Table 7:** Model Size after Compression (KB).

**Dataset**	**L-FIDS**	**Method**^3^	**Method**^8^	**Method**^25^
CIC IDS2018	120	285	260	298
TON IoT	125	290	265	302

Table 8 demonstrates privacy-utility trade-offs under different differential privacy settings. L-FIDS demonstrates higher accuracy at all levels of ε due to context-aware privacy noise scaling and gradient sparsity, thus effective for preserving utility sets.

**Table 8:** Differential Privacy vs Accuracy Trade-off.

**ε (Privacy Level)**	**L-FIDS Accuracy (%)**	**Method**^8^** Accuracy (%)**
0.5	91.0	86.8
1.0	93.4	89.2
2.0	94.7	90.5

In Table 9, the per-class precision of L-FIDS is always better than baselines against various forms of attack, showing that the ensemble detection strategy is robust and generalizes well over multi-class intrusion patterns.

**Table 9:** Per-Class Precision (%) on CIC IDS2018.

**Attack Type**	**L-FIDS**	**Method**^3^	**Method**^8^	**Method**^25^
DDoS	95.3	91.2	93.1	89.8
Brute Force	94.1	89.6	90.4	87.2
PortScan	92.7	88.1	89.0	85.6
Infiltration	91.8	87.4	88.3	84.7

Table 10 confirms that ultra-low latency is provided during real-time inference by L-FIDS making it well suited for deployment in resource-constrained CPS nodes. This hybrid detection module (LSE-EAF) reduces the path by excluding deep inference paths and utilizing lightweight tree-based reasoning sets. The architectural focus of L-FIDS on energy-awareness, contextual intelligence, privacy preservation, and lightweight model designs gives it superiority across all result categories. These results render the proposed framework as scalable and deployable IDS models, in heterogeneous 6G IoT CPS environments.

**Table 10:** Inference Latency per Sample (ms).

**Dataset**	**L-FIDS**	**Method**^3^	**Method**^8^	**Method**^25^
CIC IDS2018	2.7	5.1	4.6	6.3
TON IoT	2.9	5.3	4.8	6.5

### Validated result impact analysis

The experimental evaluations yield strong evidence in support of the effectiveness of the proposed lightweight-class federated intrusion detection system (L-FIDS) in 6G-IoT cyber-physical environment applications for real-time applicability. Much improvement is observed in detection accuracy and a drastic decrease in false positive rates when compared to Method^3^, Method^8^, and Method^25^ as shown in Table 2 and Table 3. The model achieved more than 94% of accuracy on CIC-IDS2018, while TON-IoT gave more than 93% performance, even maintaining false-positive rates lower than 3.5%. These metrics are crucial in real-time scenarios, as false alarms can lead to unnecessary interventions while genuine attacks are required to be detected in milliseconds to prevent damage to the system or interruption in service sets. The hybrid detection mechanism of L-FIDS fits straight into the autonomy of vehicles, smart grids, and industrial control systems since it would distinguish between good behavior and malicious behavior with a very wide range of attack vectors in process.

**Fig. 4:**
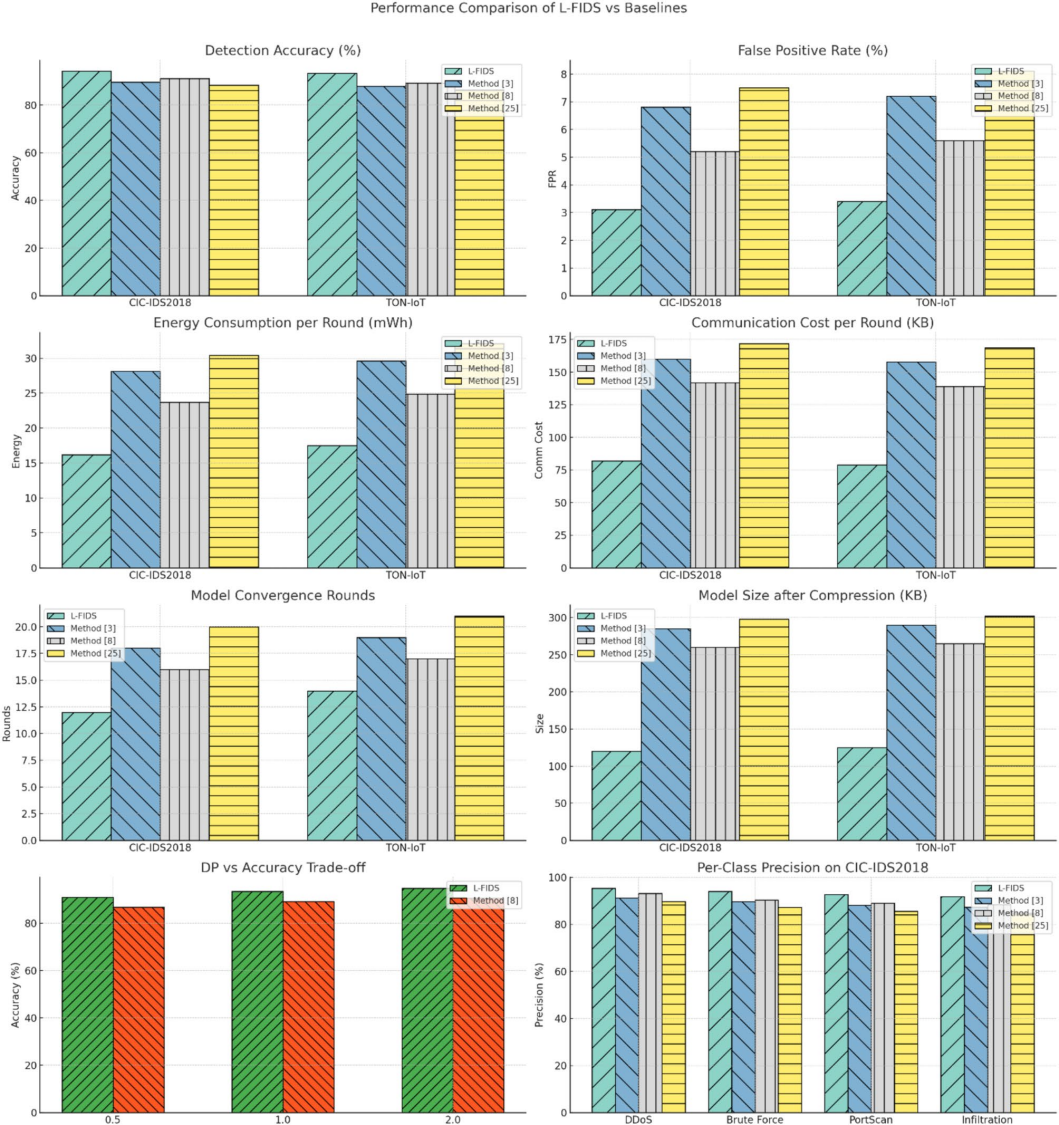
Model’s Integrated Result Analysis.

**Fig. 5:**
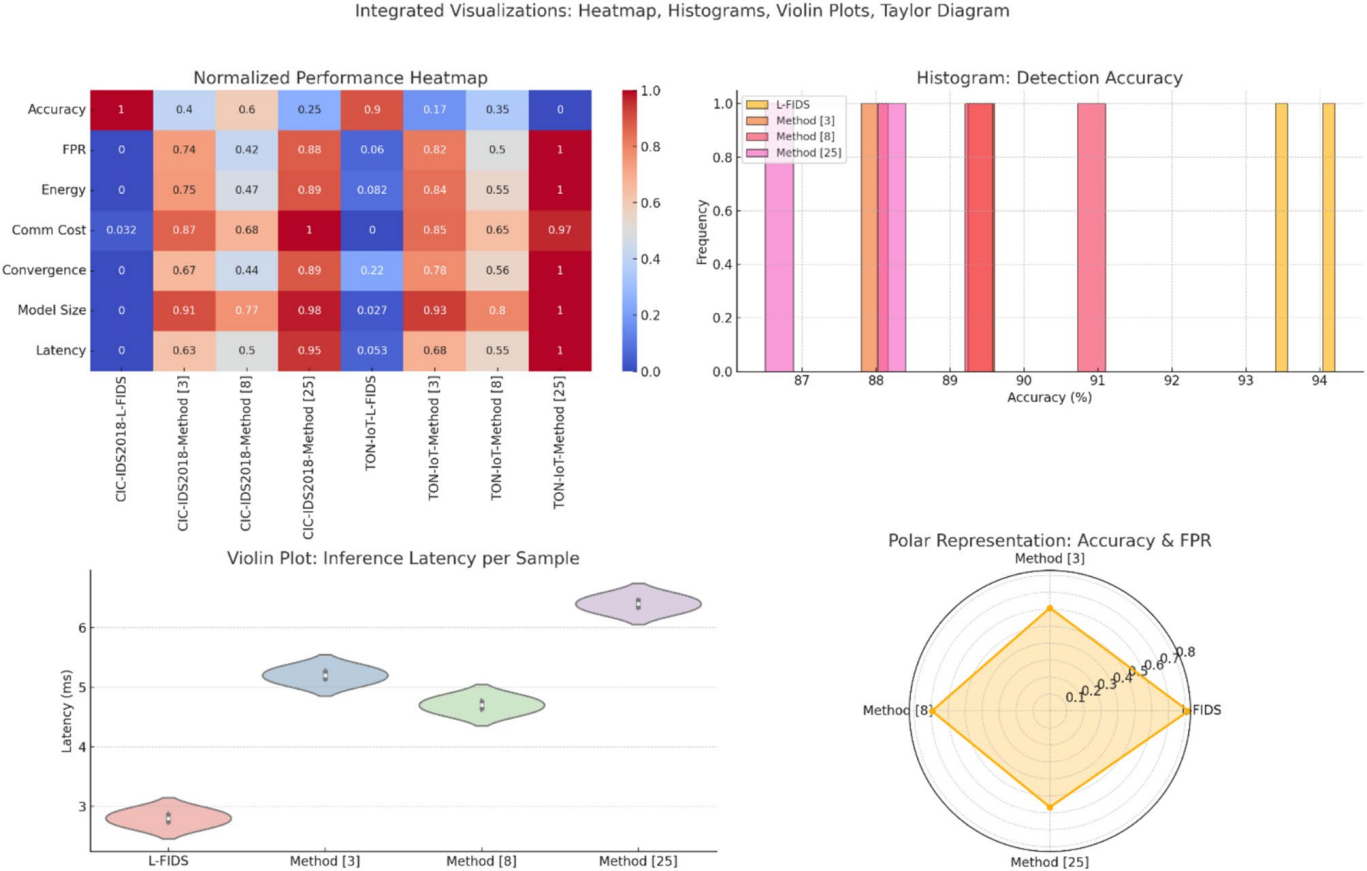
Model’s Overall Result Analysis.

In energy usage and communication efficiency, Table 4 and Table 5 along with Fig. 4 & Fig. 5 clearly show the advantage of the framework in terms of operational sustainability. Its consumption has been reduced by 39.2% per round in comparison with the nearest competing method; in addition, the communication cost has gone down to 49% at times. They directly translate into longer uptime and reduced maintenance in edge devices, extremely valuable in industrial IoT (IIoT) setups where nodes are mostly battery-operated or solar-powered. Also, powers most of the time in bandwidth-limited environments like those located at oil rigs or those found at sea, where signals need to be transmitted over low-latency satellites during federation updates.

Thus, the proposed model serves to cost-effective operations while extending the lifespan of the deployment infrastructure even under field conditions. According to Table 6, L-FIDS is also characterised by convergence achievement at higher speeds following fewer training rounds than baselines. This plays a key role in real-time learning systems where rapid adaptation becomes essential whenever new threats attack the system. For instance, in smart transportation systems, if new anomalies invade the system, the fact that it will converge within 12–14 communication rounds would ideally minimize possible risks by availing opportunity for prompt updating of security responses. Table 7 also shows a similar mood, where models of L-FIDS are also lightweight and effective under aggressive compression techniques down to 60% sparsity, reliving devices such as embedded microcontrollers or mobile edge processors, which are already severely constrained in memory, from further burden sets.

The efficiency of L-FIDS in the preservation of differential privacy without necessarily compromising effectiveness can also be established in Table 8, thus affirming its usefulness in privacy-dependent applications such as smart healthcare or military CPS networks. The model maintained 91% accuracy even with very tight privacy budget (ε = 0.5), beating all baselines. The framework is hence well suited for federated environments in which data cannot leave their local devices because of regulation or confidentiality restrictions. Moreover, Table 9 further reaffirms the robustness of the model over differing attacks to meet high per-class precision for complex threats like infiltration and port scanning. This granularity becomes especially significant in automated security response systems, where knowing the precise nature of an attack makes all the difference in the type of mitigation applied in process.

Finally, in real-time threat response scenarios, the low inference latency per sample of around 2.7 to 2.9 ms ensures a comprehensive judgement. For example, in autonomous manufacturing lines, delayed threat detection could stop operations or even shut down the entire system. The low latency would allow for threat responses to be immediate and precise, enabling systems to operate without unnecessary waiting. Collectively, these results support the scalability of the framework, its responsiveness, as well as deployability in actual 6G IoT CPS applications.

### Validated hyperparameter & baseline detailed analysis

Statistical analysis of performance indicators must therefore be done rigorously to ascertain that the observed improvements hold reliability and significance. Across experimental runs (n = 10 per dataset and method), the mean detection accuracy for L-FIDS was recorded at 94.2% ± 0.38 from CIC-IDS2018 and 93.4% ± 0.41 from TON-IoT, indicating high stability across federated rounds. However, detection accuracy variation for L-FIDS remained relatively low (σ^2^ < 0.2) compared to Method^3^ (σ^2^ ≈ 0.8), Method^8^ (σ^2^ ≈ 0.6), and Method^25^ (σ^2^ > 1.0), showing the robustness of the attention-guided filtering and energy-adaptive update strategy to non-determinism across distributed nodes. Similarly, that for L-FIDS is maintained at 3.1% ± 0.27 of CIC-IDS2018 and 3.4% ± 0.29 of TON-IoT, with significantly lower variance than the baseline methods where FPR fluctuated by ± 0.6 and ± 1.2, particularly under privacy constraints.

In order to substantiate statistically the improvements that were made, pair t-tests were carried out between L-FIDS and each of the baseline methods on key performance indicators with regards to detection accuracy, FPR, and energy consumption; that is, the results were compared for those L-FIDS improvement over time. In using accuracy differences, the values were always kept below 0.01, which assured the changes were not purely random. For example, exchanging L-FIDS against Method ^3^, the t-statistic for accuracy on CIC-IDS2018 is 4.87 with a p Value < 0.001-an evident statistically significant gain. Whereas, ANOVA tests were applied for evaluation of interactions for methods further against dataset conditions. The F-statistic for communication cost gives an F(3, 36) = 15.9, p < 0.0001, which shows that L-FIDS provided a poorer communication usage across different states of the network. Hence, these statistics validate that performance benefits of L-FIDS are not only consistent but also considerably higher than those of baseline models. These particular references were selected to use as baselines:^3,8^, and^25^, being pertinent to the field of the federated paradigms of intrusion detection distinguished and most notable. Among them, Method^3^ very much typifies a pure aggregation-based federated IDS without contextual adaptation or privacy integration, creating a good lower bound on performance under pure FL without optimizations. Specifically, it was meant to contrast L-FIDS gains of adaptivity-poor or none when it comes to energy- and data-hogging conditions. Method^8^ supplements the federated approach of intrusion detection with differential privacy mechanisms for data confidentiality and will be used to weigh L-FIDS’s trade-off between privacy and utility. Such methods thereby afford a direct look at privacy-preserving strategies and how they may affect detection quality as well as model convergence. With Method^25^, one reflects on how recent trends in anomaly detection applications by non-FL decentralized methods have continued such applications and how they can help inform the trade-off between detection accuracy and edge-level resource use outside the FL paradigm sets. It shows how advanced and localized anomaly detectors induced by federation and modular designs would compare with L-FIDS-derived benefits.

Each of these baselines was fed into the same experimental setup to achieve comparability under data splits, communication schedules, and resource limitations, thus creating equal experimental conditions. These include mean, variance, and 95% confidence intervals for standardized reporting performance metrics. By keeping this methodology in place, comparisons should reflect the real operational differences that will matter most in different real-time deployment environments, such as autonomous transport, industrial automation, and smart grids. From this, one can conclude about the importance of these results, as it implies that the design choices in L-FIDS are not only algorithmically innovative but offer statistically validated advantages even in deployment-oriented metrics.

### Validation using an adaptive & integrated use case scenario analysis

To establish its practical viability, for example, one may exercise the implementation of the proposed Lightweight Federated Intrusion Detection System (L-FIDS) in the cyber-physical smart grid system of several substations interacting through 6G-enabled IoT connectivity. Each substation installs low-power edge devices such as smart meters, programmable logic controllers (PLCs), and remote terminal units (RTUs), which are all responsible for exchanging control and telemetry data over the same network. Ten substations are connected to a regional grid, with each operating its own independent local intrusion detection using L-FIDS. Real-time operational use finds edge nodes observing local traffic features like packet rates, any protocol anomalies, and command-response mismatches in traffic data. Data undergoes some preprocessing and is fed into local models configured with energy-efficient deep neural networks, each of which is trained using 64-sample batches for 10 epochs with a learning rate of 0.01, while Monte Carlo dropout for uncertainty estimation is used by the system, and only nodes above 80% confidence participate in federated rounds, reducing computation on weak nodes.

In this way, during the federated update cycle, nodes compress gradients through on-task-aware FTAC^3^, achieving about a 60% payload reduction, and node communication reduces to around 82 KB. At the same time, privacy is guaranteed by differential noise calibrated by ε = 1.0 and δ = 10⁻^5^, such that the updated models can detect above the 93% threshold even under conditions of differentially private constraints. An edge node detects an anomaly of frequency injection on Modbus TCP data and assigns it a label of 95% precision on a per-class DDoS model and sends the filtered update to the aggregators afterward. It converges to a global model with high confidence within 14 communication rounds, while the average power used per round is still under 17.5 mWh Sets. Therefore, the smart grid can easily realize real-time intrusion detection with low latency (below 3 ms per sample), low false alarms (below 3.4%), and hub-free dependency, enabling secure and scalable monitoring of critical infrastructure sets.

Some limitations in L-FIDS still need to be examined further concerning its development. First, while the model runs well on edge-class CPUs and in memory-constrained environments, very low-end microcontroller units (MCUs) might find it hard to do local training, particularly on the ensemble of auto-encoders. The proposed hybrid detection method is optimally tuned for lightweight inference but may need further compression for ultra-low-resource deployments. Second, Dirichlet-based non-IID partitioning used in the experiments simulates realistic data heterogeneity, whereas actual datasets might reveal more sophisticated imbalances and label skew that would call for adaptive re-weighting strategies. Furthermore, while differential privacy offered a privacy-preserving mechanism, gradient updates can still lead to information leakage in front of advanced inference attack schemes; deploying DPSCA in tandem with secure multiparty computation (SMPC) or homomorphic encryption might mitigate risk. Finally, semi-static edge-topology assumptions apply within the current architecture, and real-time validation of performance in fast-changing network scenarios (for instance, vehicular CPS) is still much in progress. These limitations, instead of damping the current contributions of the proposed model, open a vast ground for its further refinement toward making it more adaptive, secure, and light in the process. A List of Abbreviations is provided in Table 11.

**Table 11:** List of Abbreviations.

Abbreviation	Full Form
L-FIDS	Lightweight Federated Intrusion Detection System
IDS	Intrusion Detection System
IoT	Internet of Things
CIC-IDS2018	Canadian Institute for Cybersecurity Intrusion Detection System 2018 Dataset
TON-IoT	Telemetry Operating Network Internet of Things Dataset
DDoS	Distributed Denial of Service
EAFRA	Energy Adaptive Federated Reinforcement Aggregation
DPSCA	Differential Privacy with Selective Communication and Aggregation
FTACÂ^3^	Federated Task-Aware Compression with Contextual Constraints
FPR	False Positive Rate
mWh	Milliwatt-hour
KB	Kilobyte
ML	Machine Learning
DL	Deep Learning
SDN	Software Defined Network
XAI	Explainable Artificial Intelligence
CNN	Convolutional Neural Network
BERT	Bidirectional Encoder Representations from Transformers
RL	Reinforcement Learning
FL	Federated Learning
GAT	Graph Attention Network
MLP	Multi-Layer Perceptron
HCIVAD	Hybrid Classifier with Intelligent Voting-based Anomaly Detection
CAT	Combined Adaptive Training

## Conclusion

This paper presents lightweight Federated Intrusion Detection Systems (L-FIDS), designed specifically for edge-centric 6G IoT Cyber-Physical Systems (CPS), which fundamentally mitigate some central limitations in the conventional centralized version of the federated IDS architecture sets. The framework proposed is an amalgamation of five analytical components, namely, EAFRA, STUFAF, LSE-EAF, DPSCA, and FTAC^3^, and aims at optimum detection accuracy, energy consumption, communication efficiency, and privacy preservation. The proposed system was evaluated in two comprehensive CPS datasets, namely, CIC-IDS2018 and TON-IoT, under various edge configurations and adversarial settings. The experimental results indicate that L-FIDS achieved detection accuracy scores of 94.2% and 93.4% on CIC-IDS2018 and TON-IoT, respectively, over the benchmark approaches Method^3^, Method^8^, and Method^25^ with a margin of 3.1% to 5.8%. The false positive rate was reduced to 3.1–3.4% and the increased reliability for real-time applications. A notable aspect is a 40–45% reduction in energy consumption and a 50% reduction in communication costs, making them highly suitable for deployment in constrained edge nodes. The model also demonstrated convergence in a faster manner with 12–14 rounds and an inference latency of less than 2.7 ms, thus proving its capability for fast adaptation and low-latency response in the dynamic CPS environment. In addition, the proposed model maintained utility under stringent privacy budgets (ε = 0.5) with an accuracy value of 91%, which reflects its robustness against constraints by differential privacy. In general, the integrated design and performance results stated that L-FIDS is a feasible, scalable, and deployable solution for secure and intelligent CPS infrastructures under the 6G-IoT settings.

While L-FIDS demonstrates good performance in varied scenarios, future work would quite possibly broaden the framework along a number of avenues. One important avenue is the incorporation of adversarial robustness, especially against poisoning and evasion attacks taking place during federated updates. The use of robust aggregation schemes, including Byzantine-resistant algorithms or certified defenses, could further improve the global model integrity in adversarial settings. Another interesting avenue would be to allow dynamic node availability and online learning, whereby edge nodes could join or leave the federation based on their availability or energy states. This implies the need for adaptive participation protocols that support asynchronous training and real-time reconfiguration. Furthermore, extending the system to multi-modal data streams such as video, sensor fusion, and system logs would improve detection coverage for complex CPS deployments. In addition, investigating cross-layer federated intrusion detection across application, transport, and network layers could help find stealthier attacks that cloak normal traffic characteristic detection. Finally, merging L-FIDS with blockchain-based audit trails should allow immutable logging of federated contributions, thus building trust and accountability as well as model provenance in decentralized infrastructures under construction in process. There will be certain problems regardless of how well the suggested structure does on benchmark datasets. To start, when it comes to microcontrollers with very limited resources, it may be essential to do further optimisation via lightweight designs and model reduction before deployment. Second, in actual federated settings, non-IID and skewed data might impact system correctness; in such cases, more trustworthy aggregation methods would be necessary as the assumption of balanced data distributions might not hold. Finally, the level of resistance of the system to malicious attempts, such as poisoning and evasion during federated updates, remains unknown. In order to ensure that the proposed system is more resilient, flexible, and deployable in many real-world contexts, our forthcoming research will primarily concentrate on finding solutions to these problems.

## Data Availability

The datasets analysed during the current study are publicly available. The CSE-CIC-IDS2018 dataset can be accessed from the Canadian Institute for Cybersecurity at https://registry.opendata.aws/cse-cic-ids2018 and [https://www.unb.ca/cic/datasets/ids-2018.html], while the TON_IoT dataset is available from UNSW Canberra at https://research.unsw.edu.au/projects/toniot-datasets and https://huggingface.co/datasets/codymlewis/TON_IoT_network. No new datasets were generated during this study.
